# Differentiation of type 2 diabetes mellitus with different complications by proteomic analysis of plasma low abundance proteins

**DOI:** 10.1186/s40200-016-0246-6

**Published:** 2016-07-20

**Authors:** Shu-Hui Yeh, Wan-Ching Chang, Hau Chuang, Hui-Cheng Huang, Rue-Tsuan Liu, Kuender D. Yang

**Affiliations:** 1 Institute of Long-term Care, MacKay Medical College, Sanzhi District New Taipei City, 252 Taiwan; 2 Department of Medical Research, Chang Gung Memorial Hospital-Kaohsiung Medical Center, Kaohsiung, 833 Taiwan; 3 Proteomic Core Laboratory, Department of Medical Research, Kaohsiung Chang Gung Memorial Hospital, Kaohsiung, 833 Taiwan; 4 Department of Medical Research, Kaohsiung Chang Gung Memorial Hospital, Kaohsiung, 833 Taiwan; 5 Division of Endocrinology & Metabolism, Chang Gung Memorial Hospital-Kaohsiung Medical Center and Chang Gung University, Kaohsiung, 833 Taiwan; 6 Department of Research & Development, MacKay Memorial Hospital, Taipei 104, New Taipei City, 252 Taiwan; 7 Department of Medicine, MacKay Medical College, New Taipei City, 252 Taiwan

**Keywords:** Type 2 diabetes, Plasma proteome, Nephropathy, Retinopathy, Albuminuria, Low abundance proteins

## Abstract

**Background:**

Few biomarkers of type 2 diabetes mellitus (T2DM) are replicable in the differentiation of T2DM with different complications. We aimed to identify proteomic biomarkers among T2DM patients with nephropathy or retinopathy.

**Methods:**

Plasma low abundance proteins were enriched by depletion of 14 high abundance proteins using an affinity removal system, and subjected to nanoflow liquid chromatography electrospray ionization (nano LC-ESI) mass spectrometry after a gel electrophoresis with in-gel digestion. The plasma differential proteomes between normal adults and diabetic patients were validated by another cohort of 149 T2DM patients.

**Results:**

A total of 826 proteins in plasma were consistently identified from 8 plasma samples of normal adults, and 817 proteins were consistently identified in 8 plasma samples of T2DM patients. Using the MetaCore analysis, low abundance proteins in plasma between normal adults and T2DM patients were significantly different in 5 functional pathways. Moreover, plasma prolactin-induced protein (PIP), thrombospondin-2 (THBS2), L1 cell adhesion molecule (L1CAM) and neutrophil gelatinase-associated lipocalin (NGAL) levels were higher in T2DM patients. Further, PIP, THBS2 and NGAL were significantly higher in T2DM patients with nephropathy (albuminuria) but not in those with retinopathy, while L1CAM levels were higher in T2DM patients with retinopathy.

**Conclusions:**

This study identified that higher PIP, THBS2 and/or NGAL levels were significantly associated with nephropathy of T2DM, and higher L1CAM but normal PIP, THBS2 or NGAL was significantly associated with retinopathy of T2DM.

## Background

Different tools have been used to uncover biomarkers for the prediction of diabetes with and without complications. Determination of advanced glycation end product (AGE) which is usually associated with poor control of hyperglycemia has been used to predict diabetes with complications [1]. Personalized tracing fasting and postprandial blood sugar or glycosylated hemoglobin A (HbA1c) have long been used to reflect blood glucose control and predict prognosis of T2DM [2]. However, some HbA1c assays’ capability to classify severity of diabetic patients is still unacceptable [3]. Microalbuminuria has recently been significantly correlated with complications of T2DM [4] and serum glucose levels were positively correlated with beta2-microglobulin and TNF-α levels [5]. These findings suggest that, in addition to hyperglycemia, low levels of inflammatory and vascular insults may contribute to diabetic complications of nephropathy and/or retinopathy [4, 5]. Moreover, high levels of homocysteine have been associated with macrovascular and microvascular complications of T1DM, but not T2DM [6], suggesting different types of diabetes and complications have different biomarkers.

Recently, proteomic approaches that identify biomarkers have been increasingly used to predict diabetes with different complications. Using ProteinChips assay, some peptides profiles in urine and blood have been referred to as biomarkers of T2DM [7, 8]. This solid-phase based mass spectrometry is usually displayed by profiles, but not specific or quantitative markers. There is also another liquid-phase bead-based proteomic approach used to differentiate T2DM patients from normal adults [9]. Although these proteomic approaches are increasingly used to search for diabetic biomarkers in blood, no significant breakthroughs have been reported [10]. This is usually limited by interference of high-abundance proteins in blood. We have previously defined T2DM with nephropathy by albumin excretion-creatinine ratio (AER) and analyzed prevalence and the risk factors for the complication [11], and defined T2DM with retinopathy by photography and assessed the correlation of the retinopathy to cardiovascular autonomic scoring scale [12]. In an attempt to identify unique biomarker(s) for the T2DM with nephropathy or retinopathy using the same cohort samples, we employed a proteomic display to compare low-abundance proteins of plasma samples between normal adults and T2DM patients, and sought to investigate whether T2DM patients with nephropathy or retinopathy had unique proteomic markers.

## Methods

### Study design and subjects studied

**Fig. 1:**
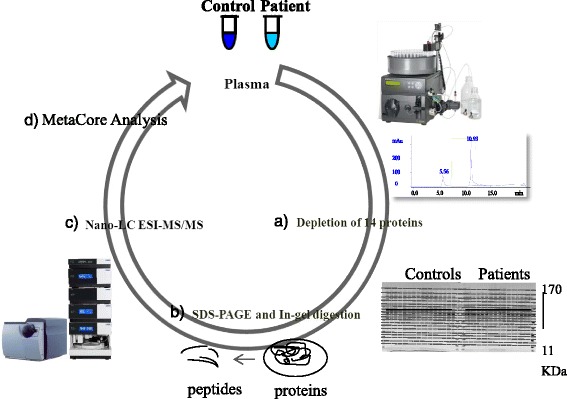
Proteomic differential displays of plasma between normal adults and T2DM patients. **a** the low abundance proteins enriched by the multiple affinity removal of 14 high abundance proteins; **b** the low abundance proteins on the SDS-PAGE gels were incited into 16 pieces for in-gel digestion; **c** each fraction of in-gel digestion was subject to nano LC-ESI analyses of peptides fingerprints; and **d** proteins identified in all 8 plasma samples were analyzed by MetaCore analysis

An age-matched case-control study design was initially used to screen proteomic differential profiles between diabetic patients and normal adults, followed by a quantitative validation of the inflammation-related differentially displayed proteins. The study was approved by the Institutional Review Board of the study hospital and informed consent was obtained from all participants. T2DM patients were recruited in the outpatient clinic of the study hospital, and normal adults who have a normal fasting blood sugar level were recruited from the Center for Health Examination of the study hospital. Plasma samples were collected in the morning with an overnight fasting condition and were harvested by ethylenediaminetetraacetate (EDTA) anticoagulant tubes. Comparison of the plasma proteomes between 8 pairs of age-matched T2DM patients and normal adults was performed by enrichment of low abundance proteins, followed by a gel-electrophoresis with in-gel digestion for nanoflow liquid chromatography electrospray ionization (nano LC-ESI) analyses and MetaCore analyses (Fig. 1). The different displays of plasma proteins were validated in another cohort of 149 T2DM patients with nephropathy or retinopathy and 30 normal adults as a control group. Normal adults were recruited from the Center for Health Examination of the study hospital who have a normal fasting blood sugar level < 105 mg/dl, normal urine analysis and normal blood pressure at systolic BP < 140 mmHg and diastolic BP < 90 mmHg.

### Definition of T2DM with nephropathy or retinopathy

Definition of T2DM with nephropathy was referred to albumin excretion-creatinine (AER) ratio in urine specimen. Albumin and creatinine concentrations were determined by nephelometry (Dade-Behring, Marburg, Germany), and nephropathy was defined as an AER exceeding 0.3 mg/mg in two urine samples collected within a 3- to 6-month period as our previously described [11]. Definition of retinopathy was determined through fundus photography, equivalent to the scale of Early Treatment of Diabetic Retinopathy Study [ETDRS] level > 20, by an ophthalmologist who was blinded to the proteomic displays of T2DM with and without complication [12]. To derive a unique phenotype of T2DM with nephropathy or retinopathy for identifying its biomarkers, we studied the samples from T2DM with nephropathy at the AER > 0.3 mg/mg in which those with co-morbidity of retinopathy (ETDRS >20) were excluded, and studied the samples from T2DM with retinopathy (ETDRS > 20) in which those with co-morbidity of nephropathy at AER > 0.3 mg/mg were excluded.

### Enrichment of low abundance plasma proteins

Plasma samples (50 ul) from 8 pairs of age-matched normal adults and T2DM patients were subject to the multiple affinity removal of 14 high abundance proteins (albumin, IgA, IgG, IgM, antitrypsin, Transferrin, haptoglobin, fibrinogen, alpha2-macroglobulin, alpha1-acid glycoprotein, apolipoproteinA1, ApolipoproteinA2, complement C3, transthyretin), purchased from Agilent Technologies (Santa Clara, CA). The low abundance proteins were harvested from the drop through phase [13]. After protein measurement, plasma samples with 50 ug were loaded to gel electrophoresis followed by in-gel digestion for nano LC-ESI mass spectrometry.

### SDS-polyacrylamide gel electrophoresis (SDS-PAGE) and in-gel trypsin digestion for nano LC-ESI mass spectrometry

We subjected the low abundance proteins (50 ug) of 8 pairs of plasma samples from normal adults and T2DM patients to SDS-PAGE. We then excised each gel into 16 parallel pieces for in-gel digestion with trypsin at 20 ug/ul. The trypsin-digested peptides were extracted twice with 1 % trifluoroacetic acid (TFA) in 100 % acetonitrile (ACN) and loaded into a nano LC-ESI mass spectrometry (Bruker-Franzen Analytik, Bremen, Germany). Mobile phase buffer was 20 % water + 80 % acetonitrile + 0.1 % formic acid at room temperature. Loading flow was 20 μL/min and flow rate was 300 nl/min. Silica-based reversed phase column C18 (PepMap 75 μm × 15 cm, 3 μm particle size, 100 Å pore size) was used. The autoproteolysis products of trypsin (m/z 842.51, 1045.56, 2211.10) were used as internal calibrates. The precision of molecular weight estimation was less than 0.5 Dalton. Identification of peptide and protein matches was performed using Mascot software (SWISS-Prot) [14].

### Validation of the protein concentrations of the 6 proteins higher differential displays in T2DM by enzyme-linked immunoassay (EIA) assay

Plasma samples (50ul) with different dilutions as recommended by the manufacturers were added to 96-well EIA plates coated with mouse monoclonal antibodies (Abcam Inc., Cambridge, UK). The wells were incubated at 25 °C for 30 min. After washing twice, the secondary antibody of goat anti-mouse immunoglobulin antibody conjugated with horseradish peroxidase (HRP) (Santa Cruz Biotech. Inc., Santa Cruz, CA) was added to the reaction for 30 min. The reactions were visualized by OD550nm (Pierce, Rockford, IL) as previously described [15]. Concentrations of plasma proteins were determined by the interpolation of a standard curve made from a series of well-known concentrations of standard samples [15].

### Statistical methods and data analysis

The differentially displayed proteomes between 8 pairs of plasma samples from normal adults and T2DM patients were initially analyzed by MetaCore software for identifying functional pathways of the differential proteins displays. The sample sizes used to validate the differentially proteomic displays were set at ≧ 30, based on the effect size of 0.4 and power of 0.8. Statistics was analyzed by the Statistical Package for Social Sciences (SPSS Inc., Chicago) version 13.0 for Windows. The adjusted p values with multiple comparisons were corrected by Bonderroni correction.

## Results

### Demographic data of subjects providing blood samples for this study

Two different batches of plasma samples from normal adults and T2DM patients were studied. The first batch of plasma samples including 8 pairs of equal gender ratio (4:4) and age-matched plasma from normal adults and T2DM patients were subject to proteomic differential displays by enrichment of low abundance proteins followed by gel electrophoresis and nano LC/ESI spectrometric analyses. After the proteomic differential displays, we used another cohort of plasma samples including 30 from normal adults and 149 from T2DM patients [11, 12] for validation of the 6 proteins related to metabolism or inflammation which were higher in the T2DM patients in the differentially proteomic displays. The demographic data among the normal adults and T2DM patients with and without complications were not significantly different as shown in Table 1.

**Table 1:** Demographic data of normal adults and T2DM patients without and with complications

Phenotypes	Number	Male/female	Age, Mean (SD)
Normal adults	30	18/12	60.0 (2.75)
T2DM, no complication	50	34/16	60.7 (8.50)
T2DM, nephropathy	49	30/19	62.0 (10.5)
T2DM, retinopathy	50	30/19	61.6 (10.4)

Gender ratios (Chi-square, *p* > 0.05) and age distributions (student t, *p* > 0.05) are not significant among groups

### Proteomic differential displays of plasma between normal adults and T2DM patients

We found that the low abundance proteins enriched by the multiple affinity removal of 14 high abundance proteins showed no different bands of high abundance proteins. The low abundance proteins on the SDS-PAGE gels were incised into 16 pieces for in-gel digestion. Each fraction of in-gel digestion was subject to nano LC-ESI analyses of peptides fingerprints. Proteins identified in all 8 plasma samples of normal adults and T2DM patients were, respectively, listed and analyzed by MetaCore analysis. A total of 826 proteins in plasma were consistently identified from 8 plasma samples of normal adults, and 817 were consistently identified in 8 plasma samples of T2DM patients. In a cut-off of 2-fold higher or lower levels of proteins between controls and T2DM, we found that the levels of 563 proteins were not different between controls and T2DM patients. Of the 344 proteins with higher levels in controls, 121 were under-detectable in T2DM patients; and of the 340 proteins with higher in T2DM patients, 110 were under-detectable in normal adults. The differential displays of proteins could be classified into 5 functional pathways. They are receptor binding, protein binding, cytoskeletal protein binding, ATP binding and adenyl ribonucleotide binding proteins, in which the former 3 classes are higher in T2DM patients, and the latter 2 are lower in T2DM patients. One hundred and ten proteins identified in T2DM patients were undetectable in normal adults, and 121 proteins identified in normal adults were undetectable in T2DM patients (Table 2). We chose to validate the 6 proteins: Dipeptidyl peptidase-4 (DPP4), prolactin-induced protein (PIP), neutrophil gelatinase-associated lipocalin (NGAL), L1 cell adhesion molecule (L1CAM), thrombospondin-2 (THBS2), and glucagon-like peptide 1 (GLP1), related to metabolism or inflammation, which could be validated by enzyme-linked immunoassay and were higher expression in T2DM patients, for differentiation between with and without complications in another cohort of population.

**Table 2:** The proteins identified in T2DM patients or normal adults were, respectively, under-detectable in normal adults or T2DM patients

Differences	No.	Names of the proteins different between normal and T2DM
T2DM > Normal	110	ABCA7; ADCY5; ADCY5; AKD1; ALPK2; ANPRB; AP3D1; APOF; ARAP2; ARMC2; ARTN; BAZ1A; BOD1L; BRPF3; BSN; CAH14; L1CAM; CASC5; CASP5; CDHR1; CKAP5; CLCF1; CWC15; CXCL1(SDF1); DAZ2; DDX12; DDX42; DDX54; DGKZ; DIDO1; DLG1; DLGP5; DMXL2; DOCK2; DOCK3; DPP4; EDRF1; EIF3A; ELP2; EP400; EPHA8; ERBB4; ERC2; ERCC6; ERI2; EXPH5; EXTL3; EYS; FAS; FBN2; FGD2; FMN2; FOG2; FOLR2; FREM3; FYN; GMIP; GLP1; GPR98; GRIN1; GUC2D; GVIN1; HNRPM; HTRA2; IF6; ITAL; KALRN; KI67; KSR1; LAT2; LENG8; LETM1; MAST3; MBD4; MCM3; MCM5; MED1; MINT; MMP3; MRAP; MRCKB; MSH6; MUC2; MYH13; MYH4; MYH7; MYH9; MYO1A; MYOF; MYST4; NBEA; NBN; NCOA3; NFM; NGAL(LCN2); NMDZ1; NUMA1; PABP2; PARD3; PDE4A; PI4KA; PIP; RGS14; SHRM2; SPTA1; SPTA1; THBS2; TLN2; TPM4; USH2A.
Normal > T2DM	121	ABCA5; ACBG1; ACSA; ACSF4; ACTS; ADNP; AIFM2; AKAP6; AL1L1; AMPM1; ANGT I; ANGT II; ANGT III; AGNT IV; POC1; APOD; ASH1L; ASXL3; ATAD5; ATRX; BAI1; BDP1; BLVRB; BPNT1; BRE1A; CAPS1; CASP; CAYP1; CCDC6; CDC5L; CDKL5; CENPJ; CHD9; CNKR2; COTL1; CP110; CROCC; CRTAP; CTC1; CUX1; DDX11; AN; DDX24; DGKD; DGKQ; DHX36; DHX9; DIAP1; DNMT1; DSG1; DYN2; DZIP1; EIF3L; ELL; ELYS; EPIPL; ERF3B; EST1A; EVPL; FABD; FAK2; FANCM; FGD4; FGD6; FGFR1; FOXE1; FRYL; FUBP2; FYCO1; H4; HBG1; HBG2; HCK; HMGX3; IDH3A; KCNH5; KCNN2; KDM2B; KDM4A; KDM5B; KDM5D; KPCD1; KRT34; KRT81; KRT82; KRT85; LAMA3; LAMA4; LIMS1; LNX1; LRRK1; MACC1; MAP1A; MASP1; MED16; MLX; MPDZ; MTUS2; MYH14; MYH3; MYH6; MYO5B; MYRIP; NBR1; NCF2; NCK2; NF1; NIN; NIPBL; NOM1; NSF; ORC1; p110 CUX1; PANK2; PB1; PERF; PEX1; PTPRT; SHRM3; SOS2; TIAM2.

### Validation of protein differential displays among T2DM patients with and without complications

Validation of the 6 proteins showing higher expression in T2DM patients on the protein differential displays was performed. We found that the plasma prolactin-induced protein (PIP) levels were higher in T2DM patients without complication (*p* = 0.020), particularly in T2DM patients with albuminuria (*p* = 0.010) (Table 3). Interestingly, T2DM patients with retinopathy had no significant increase of PIP levels. We could not validate that GLP1 (Table 4) and DPP4 (Table 5) levels were different between T2DM patients and normal adults in the EIA assays. However, we did find that thrombospondin-2 (THBS2) levels were significantly higher in T2DM patients with nephropathy but not retinopathy (Table 6). Results also showed that L1CAM levels were significantly higher in T2DM with retinopathy but not significantly higher in T2DM without or with nephropathy (Table 7) than in normal adults. In contrast, NGAL levels were significantly higher in T2DM patients with nephropathy but not retinopathy (Table 8).

**Table 3:** Plasma PIP levels among normal adults and T2DM patients with and without complications

Subjects	*n*	*M (SD)*	*p* value
Normal adults	30	249.61 (159.02)	-
T2DM, no complication	50	425.67 (473.29)	0.020
T2DM, nephropathy	49	534.32 (725.84)	0.010*
T2DM, retinopathy	50	318.64 (459.78)	0.430

*p < 0.013 were considered significant difference based on the null hypothesis of *p* < 0.05 with Bonferroni correction

**Table 4:** Plasma GLP1 levels among normal adults and T2DM patients with and without complications

Subjects	*n*	*M (SD)*	*p* value
Normal adults	30	35.20 (12.47)	-
T2DM, no complication	50	44.54 (36.17)	0.177
T2DM, nephropathy	49	37.82 (27.73)	0.627
T2DM, retinopathy	50	32.39 (34.14)	0.666

**Table 5:** Plasma DPP4 levels among normal adults and T2DM patients with and without complications

Subjects	*n*	*M (SD)*	*p* value
Normal adults	30	168.44 (40.59)	-
T2DM, no complication	50	161.74 (50.97)	0.540
T2DM, nephropathy	49	162.13 (54.47)	0.590
T2DM, retinopathy	50	166.28 (41.83)	0.370

**Table 6:** Plasma THBS2 levels normal adults and T2DM patients with and without complications

Subjects	*n*	*M (SD)*	*p* value
Normal adults	30	16.05 (5.43)	-
T2DM, no complication	50	14.29 (7.85)	0.285
T2DM, nephropathy	49	29.96 (17.20)	0.012*
T2DM, retinopathy	50	17.51 (14.93)	0.532

*p < 0.013 were considered significant difference based on the null hypothesis of *p* < 0.05 with Bonferroni correction

**Table 7:** Plasma L1CAM levels normal adults and T2DM patients with and without complications

Subjects	*n*	*M (SD)*	*p* value
Normal adults	30	0.00 (undetectable)	-
T2DM, no complication	50	1. 96 (6.41)	0.195
T2DM, nephropathy	49	2.62 (7.50)	0.018
T2DM, retinopathy	50	2.24 (6.04)	0.012*

*p < 0.013 were considered significant difference based on the null hypothesis of *p* < 0.05 with Bonferroni correction

**Table 8:** Plasma NGAL levels among normal adults and T2DM patients with and without complications

Subjects	*n*	*M (SD)*	*p* value
Normal adults	30	65.93 (22.88)	-
T2DM, no complication	50	97.65 (31.70)	0.000
T2DM, nephropathy	49	161.87 (205.76)	0.000
T2DM, retinopathy	50	51.90 (44.15)	0.111

## Discussion

Using the tool of proteomic differential displays, we have identified that many plasma metabolic and inflammatory proteins such as THBS2, NGAL and PIP levels might be good biomarkers for the correlation to T2DM with nephropathy. Also, T2DM patients with retinopathy have normal THBS2, NGAL and PIP levels but elevated L1CAM levels. Whether these biomarkers could be used for early prediction of T2DM with different complications in these patients requires further prospective studies.

Neutrophil gelatinase-associated lipocalin (NGAL) also called LCN2 (Lipocalin-2) has been shown to be a biomarker for acute kidney injury [16]. Recently, Lacquaniti, et al. [17] also showed that normoalbuminuric diabetic nephropathy was associated with higher plasma NGAL levels. In this study, we have further shown that T2DM patients with nephropathy (AER > 0.3 mg/mg) but not those with retinopathy (ETDRS > 20) has a significantly higher plasma NGAL. We also found that the plasma prolactin-induced protein (PIP) levels were higher in T2DM patients with nephropathy. PIP has been implicated in immunosuppressive effects [18]. This suggests that PIP may act an immunosuppressive biomarker for T2DM in response to microvascular inflammation in T2DM with nephropathy. The THBS2 levels were significantly higher in T2DM patients with nephropathy, but not significantly higher in T2DM patients with retinopathy. This is somewhat different from another report showing the overexpression of THBS2 in the vitreous body from patients with proliferative diabetic retinopathy [19]. In THBS2 knockout mice, absence of THBS2 was related to anti-angiogenesis and prolonged inflammation [20]. Taken together, these results suggest that certain blood inflammatory and angiogenic proteins may be involved in T2DM with nephropathy or retinopathy, and may be used as predictors of T2DM with nephropathy or retinopathy if further large-scale clinical investigations with longitudinal data validate the clinical relevance of these proteins as potential biomarkers.

Dipeptidyl peptidase-4 (DPP4) is an enzyme that could catabolize GLP1 and is implicated in T2DM patients with complication [21]. In an animal study, Balkan et al. [22] showed that inhibition of DPP4 increased plasma GLP1 concentrations and improved oral glucose tolerance. We had initially found that plasma DPP4 and GLP1 levels in mass spectrometric analyses were higher in 8 pairs of T2DM patients in comparison to those of normal adults. However, we found there was no significant difference in the validation by a quantitative enzyme-linked immunoassay with a larger population of 149 T2DM patients with nephropathy or retinopathy. This may be because the DPP4 and GLP1 are labile in the blood, or have different isoforms or metabolites not feasible for the measurement by enzyme-linked immunoassay.

Neural cell adhesion molecule, L1 (L1CAM) was found to be significantly higher in T2DM patients with retinopathy but not T2DM without or with nephropathy. L1CAM has been shown to induce FGF receptor-mediated neurite outgrowth [23]. Recently, it was also shown to be a urinary biomarker in obstructive nephropathy [24]. In blood, we are the first to show that a significantly higher L1CAM, but normal PIP, THBS2 or NGAL level was associated with retinopathy of T2DM.

The strength of this study is to deplete 14 high abundance proteins using an affinity removal system before a gel electrophoresis followed by nano LC-ESI mass spectrometry, and to validate the proteomic differentiation of T2DM patients with nephropathy or retinopathy by another cohort of 149 T2DM patients. There are also some limitations in this study. 1) Although we found 340 plasma proteins had higher levels in T2DM patients than controls, we just validated 6 of them related to metabolism or inflammation in a quantitative measurement due to limited availability of quantitative measurement of blood proteins identified in the proteomic differential displays. 2) Since this study is done in a single population recruited from a medical center, the results from this study may need further validation from other ethnic populations. 3) The quantitative validation of plasma biomarkers in the study is separately performed in an EIA. Recently, there are new complementary approaches for the mass spectrometry of high-throughput monitoring of protein modifications and the absolute quantification of proteins in a picomolar range [25].

## Conclusions

We used nano LC-ESI mass spectrometry with in-gel digestion after a gel electrophoresis for analyses of differential displays on plasma low abundance proteins between T2DM patients with and without complications, and found certain proteins were suitable for differentiation of T2DM patients with and without complications. THBS2, NGAL and PIP levels might be good biomarkers for the association of T2DM with nephropathy. Also, T2DM patients with retinopathy have normal THBS2, NGAL and PIP levels but a significantly higher L1CAM level. Further prospective studies to validate these proteins with and without modifications suitable for the prediction of T2DM patients with nephropathy or retinopathy are required.

## Abbreviations

AER, albumin excretion-creatinine; DPP4, Dipeptidyl peptidase-4; EIA, enzyme-linked immunoassay; ETDRS, Early Treatment of Diabetic Retinopathy Study; GLP1, glucagon-like peptide 1; L1CAM, L1 cell adhesion molecule; nano LC-ESI, nanoflow liquid chromatography electrospray ionization; PIP, prolactin-induced protein; NGAL, neutrophil gelatinase-associated lipocalin; T2DM, type 2 diabetes mellitus; THBS2, thrombospondin-2

## References

[1] Monnier VM, Sun W, Sell DR, Fan X, Nemet I, Genuth S (2014). Glucosepane: a poorly understood advanced glycation end product of growing importance for diabetes and its complications. Clin Chem Lab Med.

[2] Keramati T, Razi F, Tootee A, Larijani B (2014). Comparability of hemoglobin A1c level measured in capillary versus venous blood sample applying two point-of-care instruments. J Diabetes Metab Disord.

[3] Razi F, Nasli Esfahani E, Rahnamaye Farzami M, Tootee A, Qorbani M, Ebrahimi SA, Nahid M, Pasalar P (2015). Effect of the different assays of HbA1c on diabetic patients monitoring. J Diabetes Metab Disord.

[4] Sjöblom P, Nystrom FH, Länne T, Engvall J, Östgren CJ (2014). Microalbuminuria, but not reduced eGFR, is associated with cardiovascular subclinical organ damage in type 2 diabetes. Diabetes Metab.

[5] Raikou VD, Kyriaki D (2015). The relationship between glycemic control, beta2-microglobulin and inflammation in patients on maintenance dialysis treatment. J Diabetes Metab Disord.

[6] Agulló-Ortuño MT, Albaladejo MD, Parra S, Rodríguez-Manotas M, Fenollar M, Ruíz-Espejo F (2002). Plasmatic homocysteine concentration and its relationship with complications associated to diabetes mellitus. Clin Chim Acta.

[7] Merchant ML, Klein JB (2010). Proteomic discovery of diabetic nephropathy biomarkers. Adv Chronic Kidney Dis.

[8] Wu J, Chen YD, Yu JK, Shi XL, Gu W (2011). Analysis of urinary proteomic patterns for type 2 diabetic nephropathy by ProteinChip. Diabetes Res Clin Pract.

[9] Chu L, Fu G, Meng Q, Zhou H, Zhang M (2013). Identification of urinary biomarkers for type 2 diabetes using bead-based proteomic approach. Diabetes Res Clin Pract.

[10] Ma Y, Yang C, Tao Y, Zhou H, Wang Y (2013). Recent technological developments in proteomics shed new light on translational research on diabetic microangiopathy. FEBS J.

[11] Shen FC, Chen CY, Su SC, Liu RT (2009). The prevalence and risk factors of diabetic nephropathy in Taiwanese type 2 diabetes.- a hospital-based study. Acta Nephrologica.

[12] Huang CC, Lee JJ, Lin TK, Tsai NW, Huang CR, Chen SF, Lu CH, Liu RT (2016). Diabetic Retinopathy is strongly predictive of cardiovascular autonomic neuropathy in type 2 diabetes. J Diabetes Res.

[13] Dardé VM, Barderas MG, Vivanco F (2007). Depletion of high-abundance proteins in plasma by immunoaffinity subtraction for two-dimensional difference gel electrophoresis analysis. Methods Mol Biol.

[14] Yang KD, Chang WC, Chuang H, Wang PW, Liu RT, Yeh SH (2010). Increased complement factor H with decreased factor B determined by proteomic differential displays as a biomarker of tai chi chuan exercise. Clin Chem.

[15] Yeh SH, Chuang H, Lin LW, Hsiao CY, Wang PW, Liu RT (2009). Regular Tai Chi Chuan exercise improves T cell helper function of patients with type 2 diabetes mellitus with an increase in T-bet transcription factor and IL-12 production. Br J Sports Med.

[16] Soto K, Papoila AL, Coelho S, Bennett M, Ma Q, Rodrigues B (2013). Plasma NGAL for the diagnosis of AKI in patients admitted from the emergency department setting. Clin J Am Soc Nephrol.

[17] Lacquaniti A, Donato V, Pintaudi B, Di Vieste G, Chirico V, Buemi A (2013). “Normoalbuminuric” diabetic nephropathy: tubular damage and NGAL. Acta Diabetol.

[18] Sugiura S, Fujimiya M, Ebise H, Miyahira Y, Kato I, Sugiura Y (2012). Immunosuppressive effect of prolactin-induced protein. Chem Immunol Allergy.

[19] Abu El-Asrar AM, Nawaz MI, Ola MS, De Hertogh G, Opdenakker G, Geboes K (2013). Expression of thrombospondin-2 as a marker in proliferative diabetic retinopathy. Acta Ophthalmol.

[20] Lange-Assochenfeldt B, Weninger W, Velasco P, Kyriakides TR, von Andrian UH, Bornstein P (2002). Increased and prolonged inflammation and angiogenesis in delayed-type hypersensitivity reactions elicited in the skin of thrombospondin-2-defcient mice. Blood.

[21] Ravassa S, Barba J, Coma-Canella I, Huerta A, López B, González A (2013). The activity of circulating dipeptidyl peptidase-4 is associated with subclinical left ventricular dysfunction in patients with type 2 diabetes mellitus. Cardiovasc Diabetol.

[22] Balkan B, Kwasnik L, Miserendino R, Holst JJ, Li X (1999). Inhibition of dipeptidyl peptidase IV with NVP-DPP728 increases plasma GLP-1 (7–36 amide) concentrations and improves oral glucose tolerance in obese Zucker rats. Diabetologia.

[23] Williams EJ, Furness J, Walsh FS, Doherty P (1994). Activation of the FGF receptor underlies neurite outgrowth stimulated by L1, N-CAM, and N-cadherin. Neuron.

[24] Trnka P, Ivanova L, Hiatt MJ, Matsell DG (2012). Urinary biomarkers in obstructive nephropathy. Clin J Am Soc Nephrol.

[25] Yassine H, Borges CR, Schaab MR, Billheimer D, Stump C, Reaven P (2013). Mass spectrometric immunoassay and MRM as targeted MS-based quantitative approaches in biomarker development: potential applications to cardiovascular disease and diabetes. Proteomics Clin Appl.

